# Nutrition, Sedentary Behaviour and Physical Activity in Undergraduate Students: A Scoping Review with PRISMA-ScR Checklist

**DOI:** 10.3390/nu18182947

**Published:** 2026-09-08

**Authors:** Mariasole Antonietta Guerriero, Rita Polito, Nicola Mancini, Antonietta Monda, Antonietta Messina, Maria Ruberto, Salvatore Allocca, Marco La Marra, Pasquale Perrone, Girolamo Di Maio, Maria Casillo, Mario Ruggiero, Yuri Russo, Giovanni Messina, Marcellino Monda, Vincenzo Monda, Filomena Mazzeo, Maria Giovanna Tafuri, Fiorenzo Moscatelli

**Affiliations:** 1 Department of Humanistic Studies, University of Foggia, 71121 Foggia, Italy; mariasole.guerriero@unifg.it; 2 Department of Psychology and Health Sciences, Pegaso Telematic University, 80143 Naples, Italy; rita.polito@unipegaso.it (R.P.); pasquale.perrone@unipegaso.it (P.P.); girolamo.dimaio@unipegaso.it (G.D.M.); 3 Department of Education and Sport Sciences, Pegaso Telematic University, 80143 Naples, Italy; nicola.mancini@unipegaso.it (N.M.); maria.ruberto@unipegaso.it (M.R.); maria.casillo@unicampania.it (M.C.); fiorenzo.moscatelli@unipegaso.it (F.M.); 4 Department of Human Science and Quality of Life Promotion, San Raffaele Telematic University, 00166 Rome, Italy; antonietta.monda@uniroma5.it; 5 Department of Precision Medicine, University of Campania “Luigi Vanvitelli”, 80138 Naples, Italy; antonietta.messina@unicampania.it; 6 Unit of Dietetics and Sports Medicine, Section of Human Physiology, Department of Experimental Medicine, University of Campania “Luigi Vanvitelli”, 80138 Naples, Italy; salvatore.allocca@unicampania.it (S.A.); marco.lamarra@unicampania.it (M.L.M.); giovanni.messina@unicampania.it (G.M.); marcellino.monda@unicampania.it (M.M.); 7 Department of Medical, Human Movement and Well-Being Sciences, University of Naples Parthenope, Via Medina 40, 80133 Naples, Italy; mario.ruggiero@phds.uniparthenope.it; 8 Department of Law, Economics and Human Sciences (DiGiES), Mediterranea University of Reggio Calabria, Via dell’Università, 25, 89124 Reggio Calabria, Italy; yuri.russo@unirc.it; 9 Department of Economics, Law, Cybersecurity and Sports Sciences, University of Naples “Parthenope”, 80131 Naples, Italy; vincenzo.monda@uniparthenope.it; 10 Department of Literary, Linguistic and Philosophical Studies, Pegaso University, 80143 Naples, Italy; mariagiovanna.tafuri@pegaso.it

**Keywords:** sedentary behaviour, physical activity, dietary habits, diet quality, university students

## Abstract

Background/Objectives: University students are particularly vulnerable to unhealthy lifestyle behaviours, including poor dietary habits, insufficient physical activity, and prolonged sedentary time. While these three behaviours have each been extensively studied individually, the extent to which they have been jointly investigated as distinct constructs in the same undergraduate populations remains unclear. This scoping review aimed to map the available evidence jointly addressing dietary habits, sedentary behaviour, and physical activity among undergraduate university students. Methods: Following the PRISMA-ScR guidelines, a systematic search was conducted on PubMed and Scopus (search date: 27 June 2026), covering records published between 2014 and 2026 in English. Studies were eligible if they measured all three constructs (diet, sedentary behaviour, and physical activity) as distinct outcomes in undergraduate populations aged 18 years or older. Results: Of 1910 records identified, 70 studies met the eligibility criteria, comprising 62 primary studies and 8 ongoing study protocols. The primary studies spanned 26 countries across five continents, and most used cross-sectional designs; only one intervention study was identified. Across studies, inadequate diet, low physical activity, and elevated sedentary time were common and frequently co-occurred within individuals, with associations reported for mental health, body image, and weight-related outcomes. Sedentary behaviour was the construct most often absent from otherwise relevant studies and was consistently elevated when measured. Conclusions: Across the 62 primary studies spanning 26 countries, a broadly consistent pattern of suboptimal diet, insufficient physical activity, and elevated sedentary time was observed among undergraduate students.

## 1. Introduction

The global burden of non-communicable diseases continues to increase, and physical inactivity and poor dietary habits are among the most important modifiable risk factors contributing to this trend. The World Health Organisation (WHO) has recognised sedentary behaviour and insufficient physical activity as major public health challenges, recommending that adults engage in at least 150–300 min of moderate-intensity aerobic activity per week while minimising prolonged sedentary time [1]. Despite these evidence-based recommendations, adherence remains critically low at the population level, with estimates suggesting that more than one quarter of adults worldwide do not meet the minimum recommended levels of physical activity [1].

Among the various segments of the adult population, university students represent a particularly vulnerable group. The transition from secondary school to higher education is a critical period during which lifestyle behaviours are highly malleable and may be substantially disrupted. Young adults entering university for the first time are confronted with profound changes in their daily routines, social environments, living arrangements, and self-management responsibilities. This transition is frequently associated with decreased physical activity, increased sedentary time, irregular meal patterns, and a deterioration of overall dietary quality [2]. Students living away from home may face additional challenges because they lose the structure of family-based meal routines and are exposed to environments that do not always support healthy lifestyle choices [3].

Physical inactivity among university students is a well-documented phenomenon. A large-scale cross-sectional survey conducted across 23 low-, middle-, and high-income countries estimated the prevalence of physical inactivity among undergraduate students at 41.4%, with substantial variation across countries and demographic subgroups [4]. Sedentary behaviour is also highly prevalent in this population, as students spend a considerable proportion of their day in sedentary academic activities, including attending lectures, studying at desks, and using screens at home or in libraries [5]. Prolonged sedentary time has been independently associated with increased cardiometabolic risk, poorer mental health outcomes, and reduced overall well-being, even among individuals who otherwise meet recommended physical activity levels [1].

At the same time, the dietary habits of university students remain a growing concern in public health research. Studies consistently report low adherence to dietary guidelines in this population, characterised by insufficient consumption of fruits, vegetables, whole grains, and dairy products, alongside elevated intake of ultra-processed foods, sugar-sweetened beverages, and fast food [2]. These patterns appear to be shaped by multiple factors, including financial constraints, limited cooking skills and time, easy access to unhealthy food options on campus and in student neighbourhoods, and academic stress [2]. Poor diet quality during the university years not only increases the short-term risk of weight gain and metabolic alterations but may also establish long-term dietary patterns that persist into adulthood, with significant implications for the prevention of chronic disease [2,6].

The co-occurrence of sedentary behaviour, poor dietary habits, and low physical activity may create a compounded health risk that requires targeted and coordinated attention. While the literature has extensively explored physical activity, sedentary behaviour, and dietary habits as separate domains, evidence mapping the extent to which these three dimensions of student lifestyle have been jointly investigated remains fragmented and heterogeneous. Physical activity-based interventions targeting university students have demonstrated some effectiveness in improving body composition and health-related outcomes [5]; however, it remains unclear how consistently sedentary behaviour has been measured as a distinct construct alongside dietary habits and physical activity in this literature.

Previous work by our research group has contributed to mapping specific aspects of this broader issue. Guerriero and colleagues conducted a scoping review documenting the associations between sedentary lifestyle, physical activity, and psychological stress in university students, highlighting the detrimental consequences of physical inactivity on mental well-being and the need for more structured promotional strategies in academic contexts [7]. However, no comprehensive scoping review has yet mapped studies that jointly address dietary habits, sedentary behaviour, and physical activity as three distinct constructs in undergraduate university populations, or described the designs, populations, and measurement approaches used in this evidence base.

Given the heterogeneity of study designs, populations, and outcome measures present in this literature, a scoping review represents the most methodologically appropriate approach to addressing this gap. Scoping reviews, as conceptualised by Arksey and O’Malley and later refined through the Preferred Reporting Items for Systematic Reviews and Meta-Analyses extension for Scoping Reviews (PRISMA-ScR), are well suited to mapping broad evidence bases, identifying key research gaps, and synthesising findings across diverse study types [8,9].

Therefore, this scoping review aims to systematically map the available evidence that jointly addresses dietary habits, sedentary behaviour, and physical activity among undergraduate university students, and to describe the characteristics of the included studies, the measurement approaches used, and the main findings reported across this body of literature.

## 2. Materials and Methods

This scoping review was conducted in accordance with the methodological framework proposed by Arksey and O’Malley and reported following the PRISMA-ScR guidelines as described by Tricco et al. [8,9]. This scoping review was registered with the Open Science Framework (OSF) under registration code 10.17605/OSF.IO/NH3SF.

### 2.1. Search Strategy

A systematic literature search was conducted to identify primary studies addressing dietary habits, physical activity, and sedentary behaviour in undergraduate university populations. Two electronic databases were searched: PubMed and Scopus. The search was conducted on 27 June 2026. Both searches used conceptually equivalent strings combining terms for the target population, dietary behaviour, physical activity, and sedentary behaviour, without restriction to intervention-related terminology.

The following search strings were used:

PubMed:

(“university students”[tiab] OR “college students”[tiab] OR “undergraduate students”[tiab] OR “undergraduate”[tiab] OR “university population”[tiab]) AND (“physical activity”[tiab] OR “exercise”[tiab] OR “sedentary behavior”[tiab] OR “sedentary behaviour”[tiab] OR “sedentary lifestyle”[tiab] OR “physical inactivity”[tiab]) AND (“nutrition”[tiab] OR “dietary habits”[tiab] OR “eating habits”[tiab] OR “food intake”[tiab] OR “diet”[tiab] OR “nutritional education”[tiab] OR “dietary behavior”[tiab] OR “dietary behaviour”[tiab]) AND (“2014”[pdat]:“2026”[pdat]) AND (English[lang])

Scopus:

TITLE-ABS-KEY((“university student*” OR “college student*” OR “undergraduate*” OR “higher education student*”) AND (“physical activity” OR “exercise” OR “sedentary behavior” OR “sedentary lifestyle” OR “sitting time” OR “screen time”) AND (“diet” OR “nutrition*” OR “eating habit*” OR “food intake” OR “dietary intake” OR “dietary habit*” OR “meal skipping”)) AND PUBYEAR > 2013 AND PUBYEAR < 2027 AND LANGUAGE (english) AND DOCTYPE (ar)

The two sources were combined for screening (N = 1910 records: PubMed n = 1428; Scopus n = 482), with duplicates identified and removed prior to screening. PubMed and Scopus were selected as the primary databases because together they provide broad, complementary coverage of the peer-reviewed biomedical, nutritional, psychological, and public health literature relevant to this topic. Scopus indexes a wider range of international journals than PubMed alone, including literature from sport sciences, psychology, and education. The limitation of restricting the search to two databases is acknowledged in Section 4.5. 

### 2.2. Eligibility Criteria

Records were eligible for inclusion if they met all of the following criteria. Population: undergraduate university or college students aged 18 years or older. For the purposes of this review, ‘undergraduate students’ is operationalised as individuals enrolled in first-cycle (bachelor’s) degree programmes at accredited higher education institutions, aged 18 years or older. Studies including mixed samples in which undergraduates constituted the majority were retained as borderline cases with explicit caveats. Records restricted to clinical or diseased populations, postgraduate-only samples, university faculty/staff, pregnant women, or general adolescent/adult cohorts not enrolled in higher education were excluded. Study design: primary interventional studies (randomised controlled trials, quasi-experimental designs, single-arm pre–post studies) or primary observational studies (cross-sectional, cohort, longitudinal, qualitative). Study protocols describing planned designs covering all three target outcomes were retained separately as ongoing studies. Reviews, meta-analyses, scoping reviews, editorials, letters, case reports, instrument-validation studies, and acute ergogenic trials were excluded. Publication date and language: published between 2014 and 2026, in English. The lower date limit of 2014 was selected to capture evidence produced after sedentary behaviour became widely recognised and operationalised as a construct distinct from physical inactivity in public health research. Outcomes: records were required to measure all three of the following as distinct constructs: dietary habits or diet quality, sedentary behaviour (sitting time, screen time, or equivalent), and physical activity. Studies measuring only two of the three constructs were excluded under this rule. Focus: studies in which disordered eating, food addiction, binge eating, or compulsive exercise were the primary outcome were excluded.

### 2.3. Screening Procedure

Title and abstract screening of all 1604 records was conducted by the lead reviewer against the eligibility criteria above. Each record received one of the following verdicts: Include (primary study meeting all criteria), Include (protocol) (study protocol meeting all criteria but without outcome data), or Exclude, with the failing criterion recorded for each excluded record (e.g., population not undergraduate, outcome constructs not measured as distinct, ineligible study design, or outside the date/language range). Given the volume of records screened, exclusion reasons at this stage were not aggregated into quantified categories; categorised counts are reported for full-text exclusions only (Section 2.4). Borderline cases—particularly records in which sedentary behaviour was implied but not explicitly named as a measured construct—were flagged for manual re-check and resolved at full-text review. Following completion of the selection process, a second reviewer verified the eligibility of all included studies, and any uncertainties identified during this verification were resolved through discussion.

### 2.4. Study Selection

**Figure 1:**
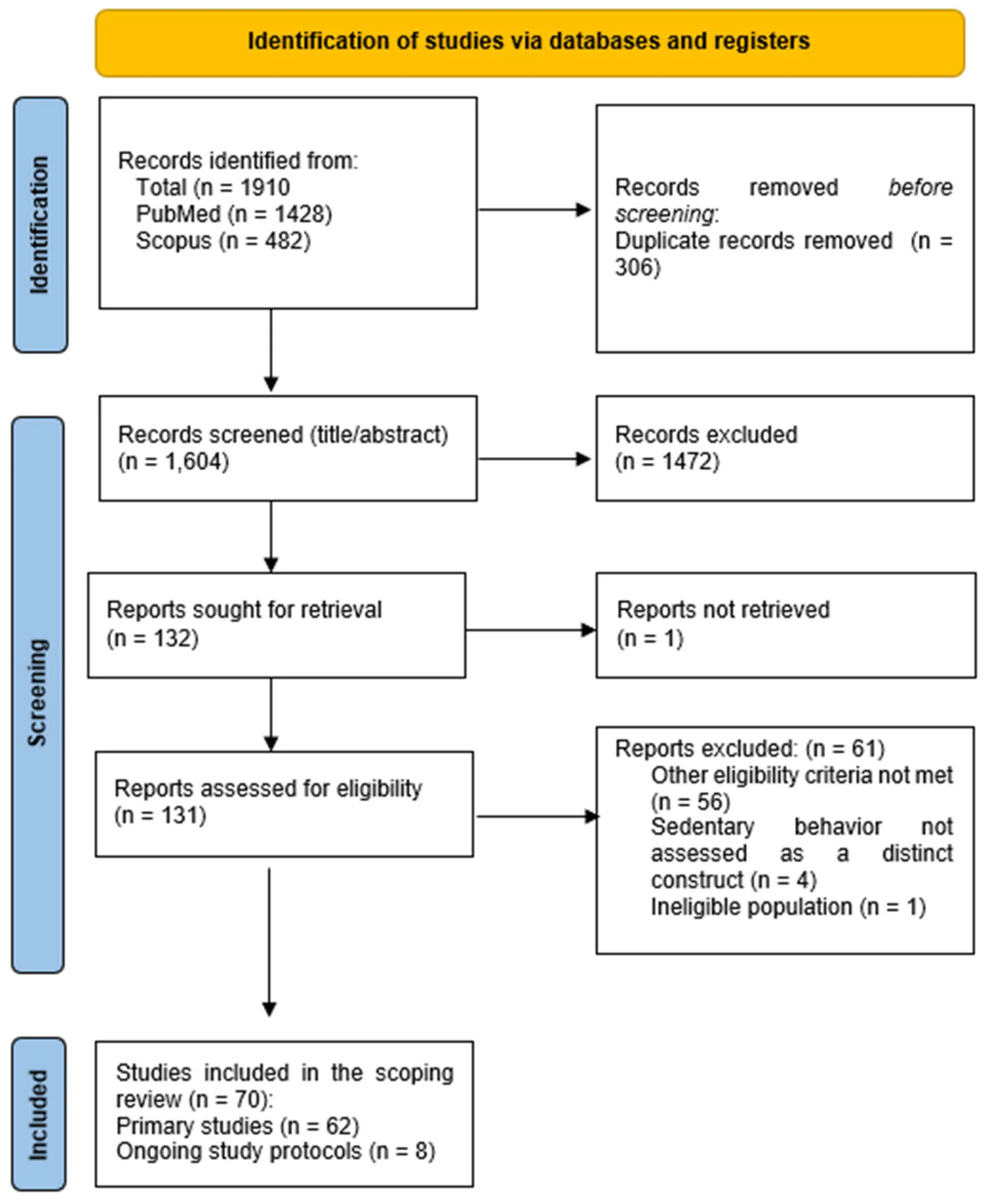
PRISMA 2020 flow diagram of the study identification and selection process.

A total of 1910 records were identified across the two databases (PubMed: n = 1428; Scopus: n = 482). After removal of 306 duplicate records, 1604 unique records were screened at the title and abstract level. Following screening, 1472 records were excluded, and 132 reports were sought for retrieval. One report could not be obtained despite attempts to retrieve the full text. Consequently, 131 full-text reports were assessed for eligibility. Sixty-one reports were excluded: 56 did not meet other eligibility criteria, four did not assess sedentary behaviour as a distinct construct, and one included an ineligible population. Seventy studies were included in the review, comprising 62 primary studies and 8 ongoing study protocols. The study-selection process is illustrated in the PRISMA 2020 flow diagram (Figure 1). The completed PRISMA-ScR checklist is provided in Supplementary File S1.

### 2.5. Data Extraction

Data extraction was performed independently by two reviewers using a standardised data extraction form developed a priori. Discrepancies were resolved through discussion and, where necessary, consultation with a third reviewer. The following information was extracted from each included study: first author and year of publication; country; study design; sample size; participants’ mean age; physical activity assessment tools; dietary assessment tools; sedentary behaviour assessment; duration (for interventional studies); primary outcomes; and main findings. The characteristics of the 70 included studies are summarised in Table 1.

## 3. Results

### 3.1. Characteristics of the Included Studies

The updated systematic search identified 1910 records, of which 70 met the strict eligibility criteria and were included in the synthesis—62 primary studies and 8 study protocols with no outcome data yet. Results from primary studies and protocols are reported separately throughout this section, as protocols provide no empirical findings and are not included in denominators used to summarise observed results.

The 62 primary studies span five continents and 26 countries. Europe is the most represented region, with studies from Spain, Italy, Germany, Poland, Sweden, Lebanon, Portugal, and the United Kingdom. Latin America contributes studies from Brazil, Chile, Ecuador, Mexico, and a multicentre study spanning ten Latin American countries. The Middle East and North Africa are represented by studies from Jordan, Saudi Arabia, Morocco, and Iran. Sub-Saharan Africa is represented by studies from Nigeria and Ethiopia. Asia and the Pacific contribute studies from Singapore, Malaysia, China, Australia, Japan, Bangladesh, and India. North America is represented by studies from the United States and Canada.

Sample sizes varied widely across the 62 primary studies, from n = 18 in a qualitative Nigerian study to n = 12,889 in a large Chinese cohort study examining weight change during COVID-19 lockdown. The median sample size across cross-sectional studies was approximately 450, although numerous studies recruited well over 1000 participants. Most participants were aged 18–25 years, with mean ages clustering between 19 and 24 years; one online-university sample was notably older, including students and staff with a mean age of 35 ± 11 years.

Among the 62 primary studies, cross-sectional designs predominated, including studies using structural equation modelling, latent profile analysis and latent class analysis. A smaller group used longitudinal, prospective, repeated-measures, qualitative, mixed-methods or diary-based approaches. One study used a single-arm pre/post wellness-coaching intervention design. The eight ongoing study protocols describe planned observational, cohort and intervention-oriented designs.

The eight ongoing study protocols were retained separately from the primary studies and are not included in the empirical synthesis. They mainly adopt longitudinal observational and cohort designs, with some planning randomised controlled trials and one developing a digital intervention tool. They were included for completeness to map forthcoming evidence in this area and will be re-examined when outcome data become available.

### 3.2. Assessment Tools

Physical activity was most frequently measured with the International Physical Activity Questionnaire, in either its short or long form, used in the majority of the 62 primary studies. The WHO Global Physical Activity Questionnaire was the second most common instrument, used in two primary studies and planned in one ongoing protocol [12,18,20]. A number of studies instead relied on bespoke self-report items adapted from established surveillance instruments such as the Health Behaviour in School-aged Children survey, VIGITEL, or the Health-Promoting Lifestyle Profile II [10,11,17,30,31,38,39,41,76]. Only a small number of studies incorporated device-based measurement, including Fitbit-based monitoring and planned accelerometry [14,24].

Diet was assessed with validated diet-quality indices in roughly half of the primary studies. The Mediterranean diet was the most common construct, captured using instruments such as PREDIMED, KIDMED, or national healthy-eating indices [18,20,26,29]. Several other validated indices were used in single studies, including a sustainable-diet score, a dietary-quality questionnaire, an eating-behaviours questionnaire, and an eating-practices scale [16,19,23,30]. The remaining studies relied on food-frequency or short dietary questionnaires, with one study capturing diet only through a single meal-skipping item, which was retained despite its limited operationalisation of dietary behaviour [10,11,17,22,25,28,31,34,38,40].

Sedentary behaviour was operationalised heterogeneously across the 62 primary studies. Sitting time derived from the IPAQ or GPAQ was the most common metric. Other studies relied on screen time or device-derived sedentary minutes from accelerometers or Fitbit devices. In one ongoing protocol, sedentary behaviour was captured within a broader campus lifestyle survey rather than with a standalone sedentary-behaviour instrument [15].

### 3.3. Prevalence of Unhealthy Lifestyle Behaviours

Across the 62 primary studies, inadequate diet, low physical activity, and high sedentary time often co-occurred among undergraduate populations. Only 8.6% of incoming Ecuadorian physiotherapy students met all three components of the Canadian 24 h movement guidelines (PA, sedentary behaviour, and sleep) at admission [81]. Similar patterns of co-occurring unhealthy behaviours were documented across multiple newly identified studies spanning Europe, Asia, Latin America, and Africa, consistently showing low physical activity, high sedentary time, and poor dietary quality in the same undergraduate populations. Among Italian students before the COVID-19 lockdown, regular physical activity adherence was already low and worsened during the lockdown, while sedentary time increased and the number of daily meals shifted upward [17]. Adherence to recommended fruit and vegetable intake was below target in Jordanian [10], Saudi [11], and Spanish [26,29] samples. Diet quality among Mexican [40] and Brazilian [30,31] undergraduates was characterised by frequent consumption of ultra-processed foods and low fruit and vegetable intake. Adherence to the Mediterranean diet was suboptimal in three Spanish samples [18,26,29].

Physical activity prevalence was more variable. In the Jordanian sample, ~81% met the WHO 150 min aerobic recommendation [10]; in the Spanish HrQoL study, most participants likewise met WHO recommendations [18]. However, low physical activity was the dominant finding in Saudi Arabia [11] and was identified as a major contributor to overweight/obesity in the Moroccan female sample (58% inactive; [76]).

Sedentary behaviour was uniformly high. Reported sedentary behaviour indicators—including sitting time and screen time as a specific subtype—included >65% of students with sitting time above recommended cut-offs in the Jordanian sample [10], 94% with risky screen use in the same sample, ~9.5 h/day of self-reported sedentary time in the Saudi sample [11], ~8.3 h/day in the Spanish HrQoL study [18], and ~10.9 h/day of total sedentary time plus ~13.4 h/day of screen time in the Singaporean Health@NUS cohort [14,34]. Sedentary behaviour was the most consistently elevated of the three constructs across studies, regardless of geography or setting. 

### 3.4. Clustering and Co-Occurrence of Behaviours

Multiple studies reported that unhealthy diet, insufficient physical activity, and prolonged sedentary time co-occurred within the same individuals, and that this clustering was associated with a greater health burden than any single behaviour alone. Barbosa et al. (2023) [30] reported that inadequate eating practices were the most common single behaviour (80.6%) and that the most common dual combination was inadequate eating plus sedentary behaviour (23.6%). Barbosa et al. (2024) [31] extended this finding by showing graded associations between the number of co-occurring obesogenic behaviours (ultra-processed-food intake, physical inactivity, and sedentary behaviour) and anxiety and depression symptoms; the dominant triad was observed in 17.2% of the n = 1353 sample. Maitiniyazi et al. (2025) [23] identified distinct latent profiles of unhealthy lifestyle clustering in Chinese college students. Domaradzki et al. (2026) [16] used structural equation modelling in a Polish sample to demonstrate that physical activity was the strongest protective factor against the fat mass index in both sexes, while a poor dietary index and, in women, sedentary time were positively associated with FMI; family affluence had no direct effect, and the models explained 30% and 37% of FMI variance in men and women, respectively. One study [19] found that, in a Spanish online-university sample (n = 2608), unhealthy behaviours clustered around poorer dietary adherence: higher physical activity and active commuting were positively associated with higher HEASUS scores, while sedentary behaviour, male sex, and overweight status were inversely associated.

### 3.5. Associations with Mental Health and Academic Outcomes

Several studies linked the diet–PA–SB triad to mental health and academic outcomes. In a German sample, healthier lifestyle behaviours were associated with better academic performance, with trait mindfulness examined as a moderator [37]. In an Australian qualitative study, students described academic performance as being influenced by a pathway in which sleep and mental well-being mediated the relationship between health behaviours, including sedentary time during online learning, and short-term academic outcomes [13]. In a Nigerian qualitative study, undergraduates identified diet, physical activity and sedentary computer/internet use as the three primary lifestyle indices shaping their health [33]. The Brazilian COVID-19-period analysis [31] demonstrated a graded relationship between behavioural co-occurrence and anxiety/depression symptoms. In Singapore, a higher count of healthy behaviours was associated with lower psychological distress and better well-being [34]. One study [32] (borderline retention) found that, in a Brazilian sample during the pandemic, lower sedentary time and dietary change reduced the odds of body-image dissatisfaction, whereas walking-based physical activity was not associated with positive body-image change.

### 3.6. The COVID-19 Inflexion

Multiple included studies were conducted partly or wholly during the COVID-19 pandemic and report on its impact on the diet–PA–SB profile of undergraduates [17,27,31,32,39,42,43,49,64]. The convergent finding across both original and newly identified studies is consistent: that the pandemic was associated with reduced physical activity, increased sedentary time, and changes in eating behaviour (typically more meals per day and increased snacking), with downstream associations with BMI increase [17], body-image dissatisfaction [32], and depression/anxiety symptoms [31]. The single intervention identified, a Behaviour Image Model brief wellness-coaching pre/post study at a US university ([39]; n = 90 completers), reported improvements in physical activity, sedentary behaviour, diet and substance use during the COVID-19 period.

### 3.7. Population Heterogeneity and Borderline Retentions

Two retained samples deviate from a strictly undergraduate-only frame: one combined bachelor’s and master’s students, while another (Giner et al., 2026) [19] included online undergraduate, master’s, postgraduate and doctoral students alongside university staff. Findings from these two studies should be interpreted as descriptive of broader university populations rather than of undergraduates only. One further study included body image as the primary outcome, which is adjacent to but not within the disordered-eating exclusion criterion; its findings are presented as behavioural associations rather than as clinical outcome evidence [32]. Two studies present additional operationalisation limits worth noting: one measured diet only as meal skipping, and another captured sedentary behaviour indirectly through the NCHA instrument set; both were retained, but readers should bear these limits in mind [15,25].

### 3.8. Ongoing Studies

The eight ongoing study protocols will collectively contribute longitudinal, multi-national, and device-augmented data on the diet–PA–SB triad in undergraduates that current evidence lacks. One protocol stands out for its scale, spanning 69 universities across 28 countries [80]. Two studies are notable for combining objective measurement, via accelerometry or Fitbit, with self-report [14,24]. One further study is among the few developing a digital intervention tool rather than a purely observational design [36]. 

## 4. Discussion

### 4.1. Principal Findings

This scoping review synthesises 62 primary studies and 8 ongoing study protocols that measure dietary habits, sedentary behaviour, and physical activity as three distinct constructs in undergraduate university populations. Two messages emerge with high consistency across diverse geographies and study designs. These findings align with the 24 h movement behaviour framework, which conceptualises physical activity, sedentary behaviour, and sleep as three interrelated and mutually compensatory components of daily time use. This integrated framework provides a theoretical foundation for the tri-construct approach adopted in the present review and underscores the importance of measuring all three dimensions simultaneously. First, the prevalence of unhealthy lifestyle behaviours is high: most undergraduates do not meet the recommended intake of fruit and vegetables, sedentary time routinely exceeds 8–10 h per day, and only a minority meet integrated movement guidelines that combine physical activity, sedentary time, and sleep targets [20]. Second, the three behaviours co-occur, and their co-occurrence carries a graded burden on mental health and weight-related outcomes [16,23,30,31,34]. Across studies that explicitly modelled co-occurrence, the dominant unhealthy combination involves prolonged sedentary time paired with poor diet, with insufficient physical activity often co-occurring.

### 4.2. Sedentary Behaviour as a Consistently Elevated Construct

The strict eligibility rule applied in this review, requiring all three behaviours to be measured as distinct constructs, highlighted an important methodological feature of the literature: sedentary behaviour was the construct most commonly missing from studies that were otherwise relevant, and this was the dominant reason for exclusion at full-text review. Where it was measured, sedentary time was consistently high across geographies, settings, and assessment modalities, including self-report, IPAQ/GPAQ sitting items, and device-derived sedentary minutes. This convergent signal—high sitting time across geographically diverse settings including Saudi Arabia, Singapore, Spain, Italy, and Jordan, among others [10,11,14,17,18,19,34]—suggests that interventions targeting only diet or only physical activity may overlook one of the most consistently elevated and potentially modifiable components of undergraduate student lifestyles.

### 4.3. The Scarcity of Interventions

A striking observation is that only one of the 62 primary studies was an intervention, a single-arm pre/post wellness-coaching study conducted during the COVID-19 period [39]. Among the eight ongoing studies, only two represented intervention-adjacent work: a large multicentre cohort with strong translational potential and a digital tool development study [36,80]. Three further intervention protocols identified during full-text screening were excluded from this synthesis because they did not meet the strict eligibility rule. The imbalance between the large body of descriptive cross-sectional evidence and the near absence of randomised intervention evidence is the principal gap in this literature. Future randomised trials that test combined diet, physical activity, and sedentary-behaviour interventions in undergraduates represent a priority research direction; where feasible, device-augmented measurement and pre-specified reporting of all three behaviours as distinct outcomes would strengthen the evidence base.

### 4.4. Mental Health, Academic Outcomes and the COVID-19 Evidence Base

A second prominent theme is the link between the diet–PA–SB triad and mental health and academic outcomes, including in qualitative work [13,33]. The COVID-19 period provided an unusual opportunity to observe changes in lifestyle behaviours under conditions of enforced environmental disruption, with multiple primary studies documenting broadly consistent decreases in physical activity, increases in sedentary time, and changes in eating behaviour [17,27,31,32,39]. This pattern strengthens the case that the lifestyle triad is sensitive to environmental disruption, although the predominantly cross-sectional and self-reported nature of the pandemic-era evidence limits causal inference.

### 4.5. Limitations and Implications for Future Research

This scoping review has several limitations that should be acknowledged. First, in accordance with PRISMA-ScR guidance, no formal critical appraisal or risk-of-bias assessment was conducted for the included studies, as this step is not a required component of the scoping review methodology and was not part of the a priori protocol. Consequently, the quality and methodological rigour of individual studies were not systematically evaluated, and the findings reported in this review should not be interpreted as indicating the strength or certainty of the underlying evidence. Following completion of the selection process, a second reviewer verified the eligibility of all included studies; this provided an additional methodological check but did not constitute fully independent dual screening of all identified records.

Second, the predominance of cross-sectional designs among the 62 primary studies limits the ability to draw causal or temporal conclusions regarding the relationships between dietary habits, sedentary behaviour, and physical activity in this population. Third, the heterogeneity of measurement instruments—particularly for dietary assessment, where tools ranged from validated food frequency questionnaires to single-item meal-skipping indicators—limits comparability across studies and precludes any quantitative synthesis. A further limitation concerns the predominance of self-reported measurement across all three constructs. Self-report instruments for physical activity and sedentary behaviour are subject to well-documented recall bias and social desirability bias, which may lead to overestimation of physical activity and underestimation of sedentary time. Similarly, dietary assessment through food frequency questionnaires and short surveys introduces recall error. Only a small number of studies incorporated device-based objective measurement [14,24], limiting the precision of behavioural estimates across the evidence base. Fourth, restricting eligibility to English-language publications may have excluded relevant evidence published in other languages, potentially introducing language bias. Fifth, the heterogeneity in sample characterisation across included studies—with many failing to report sex/gender composition, academic discipline, year of study, recruitment setting, or sampling method—limits the ability to draw conclusions about subgroup differences and generalisability across student populations. Sixth, the requirement that studies measure all three constructs (diet, sedentary behaviour, and physical activity) as distinct outcomes, while methodologically necessary to address the review’s aim, may have excluded studies that addressed only two of these dimensions but otherwise contained relevant findings, as reflected in the full-text exclusions where one or more target constructs were not assessed separately. Finally, 8 of the 70 included studies were ongoing protocols without outcome data at the time of this review, meaning that a portion of the mapped evidence reflects planned rather than completed research. Three priorities emerge from this synthesis. First, undergraduate-targeted intervention studies that act simultaneously on diet, physical activity, and sedentary behaviour are needed, as current evidence remains overwhelmingly descriptive. Second, device-augmented measurement warrants broader adoption for sedentary behaviour and physical activity, given the well-documented limitations of self-report in terms of recall and social desirability bias; a small number of the included studies already model this approach [14,24]. Third, future studies should pre-specify and report all three behaviours as distinct outcomes, a methodological practice that would substantially expand the evidence base available for future synthesis. The 8 ongoing study protocols identified in this review will materially advance this evidence base once their results are published. At the university level, these findings support the development of campus-based health promotion programmes that simultaneously target all three behaviour domains. Promising strategies include environmental redesign to reduce prolonged sitting (e.g., active workstations, movement breaks during lectures), structured dietary education integrated into student health services, and accessible physical activity facilities and programming. Such multicomponent approaches, grounded in behavioural theory, represent a logical next step for translating the descriptive evidence base into practice.

## 5. Conclusions

Across 70 studies spanning 26 countries and five continents, a broadly consistent pattern of suboptimal diet, low physical activity and elevated sedentary time was observed among undergraduate students, with these behaviours frequently clustering within individuals and tracking with mental health, body image and weight outcomes. Sedentary behaviour was the most consistently elevated construct and the most commonly omitted measurement in the wider literature. The available evidence is dominated by cross-sectional self-report data; the principal research priority is randomised interventional evidence that simultaneously targets and measures all three behaviours.

## Figures and Tables

**Table 1:** Characteristics of included studies (n = 70). PS = primary study; P = protocol. Protocol studies are retained for completeness but do not contribute to the empirical synthesis.

Type	Author(s), Year	Country	Study Design	N	PA Assessment	Diet Assessment	SB Assessed	Main Findings
PS	[10]	Jordan	Cross-sectional	136	Self-report questionnaire (PA vs. WHO recommendations)	Questionnaire on dietary pattern, intake and eating behaviours	Y—sitting > 4 h/day and screen use > 2 h/day	~81% met PA recommendations, but 65% exceeded sitting, and 94% exceeded screen time; poor fruit/vegetable intake; males consumed fast food more frequently
PS	[11]	Saudi Arabia	Cross-sectional online	1112	HPLP-II PA subscale (8 items)	HPLP-II nutrition subscale (9 items)	Y—self-reported sedentary time (h/day)	Mean sedentary time 9.51 h/day; PA subscale lowest; mental health problems associated with more sedentary time
P	Azizpour et al., 2025 [12]	Iran	Cohort protocol	426	GPAQ-v2	WHO STEPS dietary section	Y—GPAQ sedentary items	Protocol—no results yet
PS	Babaeer et al., 2025 [13]	Australia	Qualitative (focus groups)	37	Not applicable (qualitative)	Not applicable (qualitative)	Y—qualitatively discussed	Multi-level influences shaped health behaviours; sleep and well-being perceived as mediators
P	Chua et al., 2024 [14]	Singapore	Cohort protocol (mHealth)	776	Fitbit + self-report PA questionnaire	hiSG app + FFQ	Y—Fitbit sedentary time	Baseline: sedentary time 10.87 h/day; low PA; suboptimal diet
P	Dahl et al., 2025 [15]	USA	Longitudinal protocol	~350–450	Campus lifestyle survey	Diet Quality Questionnaire (33 items)	Y (campus survey)	Protocol—no results yet
PS	Domaradzki et al., 2026 [16]	Poland	Cross-sectional (SEM)	418	Polish IPAQ long form	Questionnaire of Eating Behaviours (QEB)	Y—sedentary time (SITT)	PA strongest protective factor against fat mass index; sedentary time positively associated with FMI in females
PS	Ferrara et al., 2022 [17]	Italy	Repeated cross-sectional (COVID-19)	2001	HBSC-derived self-report	Self-reported eating-habits questionnaire	Y—sedentary time increased during lockdown	PA reduced; sedentary time and eating behaviours changed; BMI increased
PS	García-Pérez-de-Sevilla et al., 2021 [18]	Spain	Cross-sectional	56	GPAQ	PREDIMED	Y—sitting time from GPAQ	Mean sedentary time 8.26 h/day; negatively correlated with physical function
PS	Giner et al., 2026 [19]	Spain (online)	Cross-sectional	2608	IPAQ-SF	HEASUS diet score	Y—IPAQ sitting time	High sedentary time; low PA; diet suboptimal; older online-university sample (borderline retention)
PS	Gutiérrez-Espinoza et al., 2025 [20]	Ecuador	Cross-sectional (1-year follow-up)	NR	GPAQ	HEI	Y—sedentary time via GPAQ	PA decreased and sedentary time increased at 1-year follow-up
P	König et al., 2023 [21]	Germany	Survey protocol	NR	IPAQ	NR	Y	Protocol—no results yet
PS	Lee et al., 2026 [22]	Malaysia	Mixed-methods	NR	Self-reported PA questionnaire	Self-reported dietary habits	Y—sedentary time	Mixed-methods findings on PA, diet and sedentary behaviour
PS	Maitiniyazi et al., 2025 [23]	China	Cross-sectional (LPA)	1340	IPAQ-SF	Diet Quality Questionnaire	Y—IPAQ sitting time	Most students in high-sedentary/low-PA cluster; distinct lifestyle profiles identified
P	Nascimento-Ferreira et al., 2022 [24]	Brazil	Cohort protocol (24 h-MESYN)	NR	Accelerometry + IPAQ	FFQ	Y—accelerometry sedentary time	Protocol—no results yet
PS	Onell et al., 2025 [25]	Sweden	Prospective cohort (1-year, SUN)	3492	Validated SUN PA questionnaire	Meal-skipping frequency (borderline)	Y—sitting time items	High sedentary time at baseline; prospective lifestyle data over one year
PS	Ramón-Arbués et al., 2021 [26]	Spain	Cross-sectional	1055	IPAQ-SF	Self-reported dietary habits	Y—IPAQ sitting time	High sedentary time; poor dietary habits; PA below WHO recommendations
PS	Sidebottom et al., 2021 [27]	USA	Cross-sectional retrospective (COVID-19)	NR	Self-reported PA items	Self-reported dietary habits	Y—screen time and sedentary time	PA decreased and sedentary time increased during COVID-19; dietary changes mixed
PS	Telleria-Aramburu et al., 2022 [28]	Spain	Cross-sectional	NR	IPAQ	Spanish HEI	Y—IPAQ sitting time	Suboptimal Mediterranean diet adherence; PA below recommendations; high sedentary time
PS	Tomás-Gallego et al., 2025 [29]	Spain	Cross-sectional	1268	IPAQ-SF	Self-reported dietary habits	Y—IPAQ sitting time	Poor dietary habits and low PA; sedentary time elevated
PS	Barbosa et al., 2023 [30]	Brazil	Cross-sectional	NR	Self-reported PA questionnaire	Self-reported dietary habits	Y—sitting time	Unhealthy dietary habits and insufficient PA; sedentary time elevated
PS	Barbosa et al., 2024 [31]	Brazil	Cross-sectional	1353	Self-reported PA questionnaire	Self-reported dietary habits	Y—sitting time	Poor dietary habits; low PA; high sedentary time
PS	Cavalcante-Neto et al., 2026 [32]	Brazil	Cross-sectional (COVID-19 comparison)	NR	Self-reported PA questionnaire	Self-reported dietary habits	Y—sedentary time	PA decreased and sedentary time increased during COVID-19; dietary quality worsened
PS	Menakaya & Menakaya, 2022 [33]	Nigeria	Qualitative (interviews)	18	Not applicable (qualitative)	Not applicable (qualitative)	Y—qualitatively discussed	Barriers to healthy eating and PA identified; sedentary lifestyle normalised
PS	Zheng et al., 2025 [34]	Singapore	Cross-sectional (Health@NUS)	NR	Fitbit + modified SPS PA questionnaire	hiSG app + FFQ	Y—Fitbit sedentary time	Suboptimal diet; high sedentary time; low PA at baseline
P	West et al., 2020 [35]	Canada	Cross-sectional protocol	NR	NR	NR	Y	Protocol—no results yet
P	Napolitano et al., 2020 [36]	USA	Digital intervention development	NR	Self-reported PA (8 targets)	Self-reported diet (8 targets)	Y—sedentary behaviour target	Formative digital intervention developed; PA, diet and sedentary behaviour targets integrated
PS	Heller et al., 2024 [37]	Germany	Cross-sectional online	1049	IPAQ-SF	Self-reported dietary habits	Y—IPAQ sitting time	High sedentary time; low PA; poor diet quality
PS	Coll Campayo et al., 2026 [38]	Spain	Cross-sectional descriptive	165	Self-administered questionnaire (WHO/ENALIA)	Structured dietary questionnaire	Y—screen time (weekday/weekend)	Nutrition students healthiest; Fine Arts students highest sedentary screen time and poorest dietary habits
PS	Pfledderer et al., 2022 [39]	USA	Single-arm pre/post intervention	121	Self-reported PA (Behaviour Image Model)	Self-reported dietary habits	Y—sedentary behaviour assessed	Wellness-coaching improved PA and dietary targets; sedentary time reduced
PS	Ramírez-Díaz et al., 2023 [40]	Mexico	Cross-sectional	NR	Self-reported PA questionnaire	Self-reported dietary assessment	Y—sedentary time	Poor dietary habits and insufficient PA; high sedentary time
PS	M et al., 2023 [41]	India (Gujarat)	Cross-sectional	400	IPAQ-SF	Self-administered dietary assessment (fruit and vegetable intake; added salt)	Y—sitting time assessed within IPAQ-SF	63% had low physical activity; 85% had inadequate fruit and vegetable intake; 41% were overweight or obese; 47% had three or more behavioural NCD risk factors
PS	Thiria et al., 2022 [42]	USA (South Carolina)	Cross-sectional online survey (pre- vs. during-COVID-19 retrospective comparison)	661 (525 students + 136 faculty/staff)	Adapted Pennington Biomedical COVID-19 Survey; minutes/day of PA; Nurses’ Health Study PA Questionnaire (MET-min/week)	REAP-s (Rapid Eating Assessment for Participants—short version); frequency of restaurant meals/cooking at home	Y	Diet quality unchanged in students, improved in faculty/staff (*p* = 0.001); PA decreased in students (*p* < 0.0001), unchanged in faculty/staff; sedentary time increased in both groups; anxiety (GAD-7) increased significantly in both groups; unhealthy diet/weight gain associated with greater declines in PA and greater increases in sedentary time/anxiety
PS	Romero-Blanco et al., 2020 [43]	Spain (Ciudad Real, Castilla-La Mancha)	Observational, cross-sectional, pre–post study (two cut-off points)	213	IPAQ-SF (International Physical Activity Questionnaire—Short Form): weekly minutes of PA and sitting time	PREDIMED (Mediterranean diet adherence questionnaire, 14 items)	Y	Both weekly PA (MD −159.87 min) and weekly sitting time (MD −106.76 min) increased during lockdown; increases in PA seen in women, most study years, normal/low BMI, non-Mediterranean-diet eaters; sitting time increased across most subgroups
PS	Monteiro et al., 2019 [44]	Brazil (Midwest—Brasília)	Cross-sectional study	2163	VIGITEL-based questionnaire; PA classified as active/inactive (≥150 min/week moderate PA cut-off, WHO criteria)	VIGITEL healthy/unhealthy diet markers (fruit, vegetables, beans, full-fat milk, meat with fat, soft drinks)	Y (“sedentary” defined as <150 min/week PA)	44.2% physically inactive; women more sedentary and had higher overweight/obesity prevalence; inactive students consumed more soft drinks/fatty meat; positive association between weight status and fruit/vegetable/bean intake
PS	Harmouche-Karaki et al., 2019 [45]	Lebanon (Beirut)	Cross-sectional study (IPAQ administered twice, 1-month interval)	221	IPAQ long form (domain-specific: occupational, transport, housework, leisure PA) and sitting time (hours/day)	Six non-consecutive 24 h dietary recalls (Nutrilog software; Nutrilog SAS, Version 2.30, France)	Y	Moderate leisure PA (vs. vigorous) associated with lower per cent body fat, independent of sitting time/energy intake; housework-related PA associated with higher per cent body fat; no association with total, occupational or transportation PA
PS	Mudarra-García et al., 2025 [46]	Spain (Madrid)	Prospective, comparative, longitudinal observational study (pre- vs. during-exams)	72 (of 142 initially recruited)	IPAQ-SF (MET-min/week; low/moderate/high)	Modified KIDMED questionnaire (Mediterranean diet adherence: low/medium/optimal)	Y	Fat mass increased (25.43% → 28.79%, *p* = 0.016); muscle mass decreased (39.70 → 36.20 kg, *p* < 0.001); visceral fat increased (2.34 → 3.52, *p* < 0.001); optimal diet quality decreased (45.8% → 20.0%, *p* < 0.001); vigorous PA dropped (84.7% → 11.1% high-PA, *p* < 0.001); sitting time increased (408 → 544 min/day, *p* < 0.001); poor diet/low PA associated with worse body composition
PS	Woodman et al., 2024 [47]	Saudi Arabia (Eastern Province)	Quantitative cross-sectional study	426	ATLS questionnaire (Part B: PA/inactivity types and frequency; minutes not reliably reported)	ATLS questionnaire (Part D: dietary habits—fast food, vegetables, dairy, sugar-sweetened drinks, cakes, frequency/week)	Y (ATLS Part C: screen time)	47% normal weight, 27% overweight, 17% obese; most nutritional/PA/sedentary factors not significantly associated with obesity; among obese group, males consuming fruits/fries/cakes/sweets >3 × /week more likely obese (not seen in females); fast food effect on obesity modified by dairy intake (OR 1.68 high-dairy vs. 0.55 low-dairy, homogeneity *p* = 0.04); vegetable intake <3 × /week associated with ~1.3 × greater obesity odds (adjusted)
PS	Coppi et al., 2022 [48]	Italy (Modena, Northern Italy)	Cross-sectional, web-based survey, two time points (first wave and second wave of COVID-19)	500 (first wave); 310 (second wave, follow-up)	Self-reported frequency of exercise (occasional, 1–2 × /week, 3–4 × /week, >5 × /week); type of exercise; no intensity data collected	40-item multiple-choice questionnaire on eating/drinking habits (meals, snacks, food categories, alcohol/coffee/tea) before vs. during quarantine	Y (screen time, sofa time)	First wave: weight gain in 48.6%, unhealthy diet switch in 43%, reduced PA (70.7% women vs. 46.3% men), worsened sleep quality (67%); women more “eating to cope” (68% vs. 43.8%), men more “drinking wine to cope” (70.5% vs. 47.5%); stress correlated with eating to cope (r = 0.86) and drinking to cope (r = 0.83); second wave showed further sleep deterioration (77% vs. 67%, *p* < 0.01) and no improvement in habits
PS	Rafraf et al., 2023 [49]	Iran (Tabriz)	Cross-sectional study	220	Validated questionnaire (Chopra et al.) assessing aerobic exercise, household chores, leisure activities, sitting time, breaks from sitting, before vs. during COVID-19	Validated questionnaire assessing meal pattern regularity, balanced diet, dairy/protein intake, fast food/fried/junk food, sugar intake, sugar-sweetened beverages, before vs. during COVID-19	Y (sitting time at work, screen time)	Eating habits improved (regular meals, balanced diet, more dairy/protein, less fast food/fried/junk food/sugar, all *p* < 0.001); PA decreased (less aerobic exercise and leisure activity) while sitting/screen time increased (>5 h screen time: 17.8% → 47.8%, *p* < 0.001); sleep time increased but quality worsened (*p* < 0.001); stress/anxiety increased; 2.2% started smoking; 34.5% reported weight gain
PS	Ramón-Arbués et al., 2022 [50]	Spain	Cross-sectional	868	IPAQ—Short Version (self-reported, 3 levels: low/moderate/high)	Spanish Healthy Eating Index (SHEI), 10 items, 0–100 score	Y (screen time, hours/day)	66.2% reported positive QOL; the psychological domain scored lowest; self-esteem and sleep quality were the strongest predictors across all domains; age, BMI, alcohol/tobacco use, and physical activity level were variably associated with QOL domains
PS	Laska et al., 2015 [51]	USA (Minnesota)	Cross-sectional (pooled annual survey data 2007–2011)	33,882 (analytic sample; 33,907 initially)	YRBSS-adapted items (strenuous/moderate activity, strengthening exercise; categorised)	Adapted YRBSS items (fruit/veg, soda, fast food, restaurant, breakfast frequency)	Y (screen time: TV/video games, hours/week)	Bisexual/lesbian women had higher obesity risk; bisexual women showed highest-risk profile (diet, PA, weight control); gay/bisexual men had poorer PA patterns but lower soda intake than heterosexual men
PS	Noonil et al., 2026 [52]	Thailand (Southern)	Cross-sectional (secondary analysis)	372	Exercise (yes/no, self-reported)	Food frequency questionnaire (FFQ, 56 items/20 food groups; adapted from Thai Food Consumption Survey); PCA-derived dietary patterns	Y (yes/no self-report)	Underweight (22.0%) and EBW (26.3%) coexisted; 3 dietary patterns identified; Thai dish pattern positively linked to year of study, breakfast/supper intake in underweight group; snack pattern linked to sedentary behaviour/exercise in EBW group
PS	Alzamil et al., 2019 [53]	Saudi Arabia	Cross-sectional	456	Arab Teens Lifestyle Study (ATLS) questionnaire (validated; frequency/duration/intensity across domains)	Arab Teens Lifestyle Study questionnaire (breakfast, fruit, vegetables, milk/dairy, sugary drinks, fast food, etc., days/week)	Y (screen viewing time, hours/day; validated cutoffs)	Nearly half physically inactive; >85% sedentary >3 h/day; 95% insufficient sleep; predictors of total PA were sleep duration and low French fries/chips intake
PS	Alshehri et al., 2023 [54]	Saudi Arabia	Cross-sectional	1420 (1377 for demographic analysis)	HPLP-II Physical Activity subscale (self-reported, Likert)	HPLP-II Nutrition subscale (self-reported, Likert)	Y (single question, hours/day sitting)	LBP prevalence 66.4%; LBP associated with poor sleep quality (β = 0.2, *p* < 0.001) and sedentary duration (β = 0.03, *p* = 0.01) after adjustment; physical activity association lost significance after full adjustment
PS	Du et al., 2021 [55]	Multi-country— China, Ireland, Malaysia, South Korea, Taiwan, Netherlands, USA	Cross-sectional	2254 (completed survey; 2663 initiated)	IPAQ (total METs-min/week, self-reported)	Starting the Conversation (STC) 8-item food frequency questionnaire, dietary risk score 0–16	Y (IPAQ sitting time, min/day)	All countries reported poor sleep quality (PSQI > 5); poorer sleep quality during COVID-19 associated with higher dietary risk (*p* = 0.001); improved sleep associated with less sitting time (*p* = 0.010); USA/Ireland students most negatively affected by pandemic vs. Asian countries
PS	Hutchesson et al., 2021 [56]	Australia	Cross-sectional (secondary analysis, online survey)	1965	Active Australia Survey (min/week; categories: inactive < 150, active 150–300, highly active > 300 min/week)	NSW Adult Population Health Survey (fruit/vegetable, sugar-sweetened beverages—short questionnaire)	Y	3 behavioural classes: “healthier” (48.6%), “moderate” (40.2%), “unhealthy” (11.2%); moderate/unhealthy classes had a higher OR of moderate/high psychological distress (up to OR 11.69)
PS	Monteiro et al., 2026 [57]	Portugal	Cross-sectional observational	163	Accelerometer wGT3X-BT (5 consecutive days; Troiano 2008 cut-offs [58])	2 non-consecutive 24 h dietary recalls (USDA multiple-pass method); NOVA classification + Nutri-Score + Traffic Light	Y	UPF = 24.8% of TEI; no association between UPF-BC/PA; poorer UPF nutritional quality associated with lower fat-free mass (FFM) and higher sedentary time (ST)
PS	Caamaño-Navarrete et al., 2025 [59]	Chile	Cross-sectional	211	IPAQ-SF (short form, previous 7 days; self-reported)	15-item dichotomous (yes/no) questionnaire on dietary habits, previously validated in Chilean students	Y	Poor diet, sleep < 6 h, ST > 4 h/day and physical inactivity associated with worse QoL and lower SWL across all dimensions
PS	Peltzer & Pengpid, 2017 [60]	8 ASEAN countries (Indonesia, Laos, Malaysia, Myanmar, Philippines, Singapore, Thailand, Vietnam)	Cross-sectional multi-country	6783	IPAQ short form (7 days; low/moderate/high categories per IPAQ protocol)	2 items on fruit/vegetable servings + items on red meat consumption, breakfast, snacks, attempt to avoid fat/fibre intake (self-reported questionnaire)	Y	Frequent snacking and ‘avoiding fat’ associated with higher obesity/WHtR/WC; PA and sedentary behaviour NOT associated with any obesity indicator
PS	Dun et al., 2021 [61]	China (Zhejiang and Hunan)	Retrospective observational (dual-centre, pre–post lockdown)	12,889	Modified Chinese IPAQ-Long Form (MET-hr/week; pre- vs. during lockdown)	Questionnaire on meal frequency (breakfast/lunch), snacking, alcohol; food composition NOT assessed	Y	Mean weight gain: 2.6 kg (men), 2.1 kg (women) (+4.2% both); increased sedentary time (+3.6–3.9 h/day) and COVID-19-related stress/depression significantly associated with weight gain; PA associated with weight change in women only; diet not associated with weight change
PS	Khajuria et al., 2025 [62]	India (18 universities, North and South India)	Cross-sectional (online survey)	506	Self-reported questions on exercise/yoga frequency (never/rarely/sometimes/regularly); not a validated/standardised instrument	Self-constructed questionnaire on meal location/frequency and consumption frequency of specific food categories (fruit, vegetables, meat, snacks, etc.)	Y	Low participation in outdoor activities and physical activity/yoga; dietary habits showed moderate improvement, but stress induced overeating in some; high prevalence of elevated screen time and disturbed sleep; low alcohol/smoking use; significant correlations between healthy lifestyle habits and better psychological wellbeing, sleep quality and overall health outcomes (r up to 0.50); sex differences: men more physically active, women better diet
PS	Savage MJ, Procter EL, Magistro D, Hennis PJ, Donaldson J, Leslie-Walker A, Jones BA, James RM (2024) [63]	United Kingdom	Observational cohort (cross-sectional, pooled from 3 annual cohorts 2021–2023)	6327	Validated: International Physical Activity Questionnaire—Short Form (IPAQ-SF); MVPA, MPA, VPA, WPA in min/week	Validated: Short-Form Food Frequency Questionnaire (SFFFQ); Diet Quality Score (DQS) calculated per SFFFQ protocol	Y (IPAQ-SF sitting time on weekdays; 53.8% ≥ 6 h/day sedentary behaviour)	30% not meeting PA guidelines; 54% sedentary ≥ 6 h/day; 83% unhealthy diet; 50.5% high-risk drinking; 18% poor sleep quality; 32% overweight/obese; men had better mental health and movement/sleep behaviours than women; TGD students had poorer outcomes in most domains; minoritised ethnicity students had better movement, drinking, and sleep behaviours and better mental health than White students
PS	Li Y (2024) [64]	China	Cross-sectional	1426	Self-report, non-validated; physical activity time change during COVID-19 (reduced/basically unchanged/increased)	Self-report, non-validated; intake change in cereals, soybeans, meat, eggs, milk, vegetables, fruits, water; dietary regularity	Y (self-reported sedentary time change: reduced/basically unchanged/increased)	The weight gain group had significantly increased intake across food categories vs. other groups; decreased physical activity, depressive symptoms, and anxiety symptoms were independent risk factors for weight gain (OR = 1.643, 1.695, 1.389, respectively) after adjustment; no significant association between increased sedentary time or poor sleep quality and weight change after adjustment
PS	Li Y, Liang J, Ma J, Jia Z, Li X, Su Q (2026) [65]	China	Cross-sectional, web-based survey	9122	Validated: IPAQ-SF; MET-min/week calculated, classified as low (<600), moderate (600–2999), high (≥3000)	Self-report based on Chinese Dietary Guidelines (2022) and international FFQs; scored 0–2 per item, classified as good (≥15), moderate (10–14), poor (≤9)	Y (adapted from IPAQ and Sedentary Behaviour Questionnaire; daily/continuous sitting time; classified low/moderate/high risk)	MSP prevalence 58.7% (neck 43.6%, back 37.1%, shoulders 36.2%); 22.6% moderate and 18.5% severe pain burden; independent predictors of MSP prevalence: female gender (OR = 1.99), poor sleep (OR = 2.81), poor diet (OR = 1.83), high sedentary risk (OR = 1.33), long screen duration (OR = 1.49), low physical activity (OR = 1.14); similar pattern for pain burden; poor sleep was the strongest predictor (OR = 3.72)
PS	Bravini et al., 2022 [66]	Italy	Cross-sectional	554	Self-reported questionnaire	Nutritional knowledge and habits questionnaire	Y	30% active; sedentary lifestyle and unhealthy diet associated with overweight
PS	Yoshida A., 2026 [67]	Japan	Cross-sectional survey	339	Self-reported exercise frequency	Self-reported dietary habits	Y	51.1% of men and 68.2% of women gained weight; sedentary time predicted prolonged retention in men
PS	Zhong Q. & Huang J., 2026 [68]	NR	Diary study (EMA, 28 days)	120	Twice daily self-report	Twice daily self-report	Y	Sleep duration predicted less sedentary behaviour; positive affect mediated sleep-exercise-diet relationships
PS	Araya-Quintanilla et al., 2023 [69]	Chile	Prospective longitudinal (1-year follow-up)	376	GPAQ	Healthy Eating Index (HEI)	Y (min/week via GPAQ)	PA decreased, sedentary time increased (+45 min/week), diet quality deteriorated, weight/BMI increased
PS	Elsayed et al., 2023 [70]	Saudi Arabia	Cross-sectional (retrospective before vs. during lockdown)	528	Self-reported (binary)	Self-reported dietary habits questionnaire	Y (screen time)	35.2% gained weight; food intake increased 48.1%; screen time increased; exercise increased 26.5% to 43%
PS	Sisay, 2021 [71]	Ethiopia	Repeated cross-sectional (3 time points)	75	IPAQ (METs/week)	FFQ adapted for Ethiopian dietary practices	Y (sitting time from IPAQ)	Males had higher VO_2_max; no association between VO_2_max and dietary changes; students spent the majority of time sitting
PS	Abdul Rahman et al., 2023 [72]	Brunei	Cross-sectional (online survey)	1020	Global Physical Activity Questionnaire (GPAQ), MET-min/week, ≥600 MET-min/week = active	WHO STEPS 16-item survey (snack, sugar, salt, fruit/veg intake)	Y (self-reported sedentary hours/day)	Poor mental wellbeing 30% (higher in females); physical inactivity 42.8%; high snack/sugar/salt intake very prevalent; smoking/obesity/sweet drinks/fight sports linked to poor wellbeing; water sports protective
PS	Akter & Hossain, 2024 [73]	Bangladesh (Dhaka)	Cross-sectional (online survey, snowball sampling)	444	Structured questionnaire items (frequency: daily/infrequent/none), self-reported	Structured self-administered questionnaire (meal regularity, food preference, junk/fried food, fruit intake)	Y (TV, internet, mobile phone use hours/day)	44% irregular breakfast; only 19% ate fruit daily; 55% preferred junk food; only 19% did daily PA; significant gender differences in dinner habits, smoking, alcohol, TV, PA
PS	Zhang et al., 2024 [74]	China	Cross-sectional (online survey, convenience sampling)	1793	International Physical Activity Questionnaire—Short Form (IPAQ-SF), MVPA min/week per Canadian 24 h MG	Chinese Health-Promoting Lifestyle Profile II (HPLP II)—fruit and vegetable intake frequency (4-point Likert)	Y (IPAQ-SF sedentary behaviour item, part of 24 h MG)	Only 27.8% met all 3 movement guidelines; meeting all three was associated with higher vegetable (OR 2.41) and fruit (OR 2.05) intake; sleep guideline alone linked to higher fruit intake
PS	Benaich et al., 2020 [75]	Morocco (Rabat)	Cross-sectional (self-administered questionnaire + anthropometric measurement)	438	Arab Teens Lifestyle Study (ATLS) questionnaire; MET-min/week via Compendium of Physical Activities; WHO guideline ≥ 600 MET-min/week	ATLS-derived questionnaire; healthy (breakfast, fruit, veg, dairy) vs. unhealthy (fast food, fried potatoes, sweets, sugary drinks) food frequency	Y (screen time, hours/week watching TV/other screens)	Overweight/obesity prevalence 14.8%/1.6%; frequent fast food, fried potatoes, sugary drinks is correlated to higher odds of overweight/obesity (OR ~2–2.4); sex differences in dietary patterns and PA; screen time not clearly associated
PS	Boukrim et al., 2021 [76]	Morocco (Southern Morocco)	Cross-sectional observational study	200 (female students)	Standardised questionnaire assessing physical activity practice and frequency	Standardised questionnaire assessing cake, fast-food and sugar-rich food consumption	Y (self-reported television/screen time: <2, 2–4 or >4 h/day)	58% were physically inactive; 47% reported > 2 h/day of television time; 77% consumed cake/fast food; 21% were overweight and 3% obese
PS	Maté-Muñoz et al., 2023 [77]	Spain (Madrid)	Single-centre cross-sectional online survey	961	IPAQ-SF (MET-min/week; low/moderate/high activity)	KIDMED questionnaire (Mediterranean-diet adherence)	Y (IPAQ-SF sedentary time)	Higher PA was associated with healthier eating habits and better self-perceived health; sedentary status was negatively associated with perceived PA; smoking/alcohol and binge drinking were associated with lower Mediterranean-diet adherence
PS	Xu et al., 2017 [78]	China (six universities, Chongqing)	Cross-sectional survey	1976	Self-administered Social Cognitive Theory-based physical activity and nutrition behaviour scale	Social Cognitive Theory-based scale assessing fruit/vegetable and sugar-sweetened beverage intake	Y (daily television viewing and computer-sitting time)	60.1% exercised < 30 min/day; 16.5% spent ≥ 4 h/day watching TV or using a computer while sitting; 79% consumed < 5 cups/day of fruit and vegetables; self-efficacy predicted healthier PA and nutrition behaviours
PS	Bertrand et al., 2021 [79]	Canada (Saskatchewan)	Cross-sectional survey, retrospective (pre-pandemic) + prospective (during pandemic)	125	Godin Leisure-Time Exercise Questionnaire (GLTEQ), moderate-to-vigorous min/week vs. Canadian 24 h Movement Guidelines	Canadian Diet History Questionnaire II (CDHQII), 165-item FFQ; energy, macro/micronutrient intake via Food Processor software (Food Processor® Nutrition Analysis Software, version 11.1, di ESHA Research)	Y (self-reported daily sedentary hours vs. Canadian 24 h Movement Guidelines ≤ 8 h/day)	Nutrient/caloric intake significantly decreased during the pandemic; alcohol intake increased; PA guideline adherence dropped from 16% to 9.6%; sedentary behaviour guideline adherence dropped from 54% to 30%; sedentary hours increased from 8.3 to 11 h/day
P	Schuch et al., 2026 [80]	Multi-country—28 countries (69 universities worldwide)	Prospective multicentre worldwide cohort study protocol; baseline assessment with follow-ups at 1, 2 and 3.5 years	Planned: approximately 20,700 (300 participants per site)	U-SMILE physical activity domain (self-reported frequency in a regular week over the previous month)	U-SMILE diet and nutrition domain (self-reported frequency in a regular week over the previous month)	Y (two self-report questions assessing average daily time spent sitting and lying/reclining)	Protocol; no empirical outcomes yet; designed to examine trajectories and associations between lifestyle behaviours and mental health symptoms across the university years

## Data Availability

The data extracted from included studies are available from the corresponding author upon reasonable request.

## Supplementary Materials

The following supporting information can be downloaded at: https://www.mdpi.com/article/10.3390/nu18182947/s1, Supplementary File S1: Completed PRISMA-ScR checklist.

## References

[1] Bull F.C., Al-Ansari S.S., Biddle S., Borodulin K., Buman M.P., Cardon G., Carty C., Chaput J.P., Chastin S., Chou R. (2020). World Health Organization 2020 guidelines on physical activity and sedentary behaviour. Br. J. Sports Med..

[2] Almoraie N.M., Alothmani N.M., Alomari W.D., Al-Amoudi A.H. (2025). Addressing nutritional issues and eating behaviours among university students: A narrative review. Nutr. Res. Rev..

[3] Moscatelli F., De Maria A., Marinaccio L.A., Monda V., Messina A., Monacis D., Toto G., Limone P., Monda M., Messina G. (2023). Assessment of Lifestyle, Eating Habits and the Effect of Nutritional Education among Undergraduate Students in Southern Italy. Nutrients.

[4] Pengpid S., Peltzer K., Kassean H.K., Tsala Tsala J.P., Sychareun V., Müller-Riemenschneider F. (2015). Physical inactivity and associated factors among university students in 23 low-, middle- and high-income countries. Int. J. Public Health.

[5] Pfisterer J., Rausch C., Wohlfarth D., Bachert P., Jekauc D., Wunsch K. (2022). Effectiveness of Physical-Activity-Based Interventions Targeting Overweight and Obesity among University Students-A Systematic Review. Int. J. Environ. Res. Public Health.

[6] Mazzeo F. (2016). Current Concept of Obesity. Sport Sci..

[7] Guerriero M.A., Dipace A., Monda A., De Maria A., Polito R., Messina G., Monda M., di Padova M., Basta A., Ruberto M. (2025). Relationship Between Sedentary Lifestyle, Physical Activity and Stress in University Students and Their Life Habits: A Scoping Review with PRISMA Checklist (PRISMA-ScR). Brain Sci..

[8] Arksey H., O’Malley L. (2005). Scoping studies: Towards a methodological framework. Int. J. Soc. Res. Methodol..

[9] Tricco A.C., Lillie E., Zarin W., O’Brien K.K., Colquhoun H., Levac D., Moher D., Peters M.D.J., Horsley T., Weeks L. (2018). PRISMA Extension for Scoping Reviews (PRISMA-ScR): Checklist and Explanation. Ann. Intern. Med..

[10] Alkhalidy H., Orabi A., Alzboun T., Alnaser K., Al-Shami I., Al-Bayyari N. (2021). Health-Risk Behaviors and Dietary Patterns Among Jordanian College Students: A Pilot Study. Front. Nutr..

[11] Alothman S.A., Al Baiz A.A., Alzaben A.S., Khan R., Alamri A.F., Omer A.B. (2024). Factors Associated with Lifestyle Behaviors among University Students—A Cross-Sectional Study. Healthcare.

[12] Azizpour Y., Salehi S., Mirzad M., Farzi Y., Karimi K., Zarepour P., Behgam N., Najafi A., Razi F., Tabatabaei-Malazy O. (2025). Lifestyle and non-communicable diseases risk factors: Changes in nutrition, sleep patterns, tobacco use, mental health, and physical activity during the first three years of university: Study protocol. J. Diabetes Metab. Disord..

[13] Babaeer L.Y., Stylianou M., Nickbakht M., Gomersall S.R. (2025). A Qualitative Investigation of University Student’s Perceptions of Health Behaviours and Associations With Educational Outcomes Through the Lens of the WHO Framework on Health Behaviours and Educational Outcomes. Health Promot. J. Aust..

[14] Chua X.H., Edney S.M., Müller A.M., Petrunoff N.A., Whitton C., Tay Z., Goh C.M.J.L., Chen B., Park S.H., Rebello S.A. (2024). Rationale, Design, and Baseline Characteristics of Participants in the Health@NUS mHealth Augmented Cohort Study Examining Student-to-Work Life Transition: Protocol for a Prospective Cohort Study. JMIR Res. Protoc..

[15] Dahl A.A., Fandetti S.M., Moore-Harrison T., Barojas Chavarria R., Raya M., Racine E.F. (2025). The campus healthy lifestyles, outcomes and experiences study: Protocol for an observational assessment of college students’ health. Front. Public Health.

[16] Domaradzki J. (2026). Family Affluence and Lifestyle Behaviors as Determinants of Fat Mass Index in University Students: A Sex-Specific Structural Equation Modeling Approach. Nutrients.

[17] Ferrara M., Langiano E., Falese L., Diotaiuti P., Cortis C., De Vito E. (2022). Changes in Physical Activity Levels and Eating Behaviours during the COVID-19 Pandemic: Sociodemographic Analysis in University Students. Int. J. Environ. Res. Public Health.

[18] García-Pérez-de-Sevilla G., Pérez-Chao E.A., Pareja-Galeano H., Martínez-Jiménez E.M., De-la-Plaza-San-Frutos M., Sánchez-Pinto-Pinto B., Romero-Morales C. (2021). Impact of lifestyle on health-related quality of life among young university students: A cross-sectional study. Sao Paulo Med. J..

[19] Giner M.P., González-Serrano B.P., Pardo A., Aguilar-Martínez A., Gomez-Donoso C., Ventura P.S., Esquius L., Bosque-Prous M., Bach-Faig A. (2026). Adherence to healthy and sustainable diets and health-related behaviors in a Spanish online university setting. Front. Nutr..

[20] Gutiérrez-Espinoza H., Cassola-Cajiao M., Garzón-Ulloa E., Celi-Lalama D., Araya-Quintanilla F., Valenzuela-Fuenzalida J., López-Gil J.F. (2025). Changes in the lifestyle behavior and anthropometrics of university students after the first year: A one-year prospective observational study. Front. Sports Act. Living.

[21] König D., Jendricke P., Poggel K., Staab L., Gollhofer A. (2023). Study protocol for evaluating the current status and needs assessment of health-related characteristics among students at Albert-Ludwigs-University Freiburg. PLoS ONE.

[22] Lee K.X., Quek K.F., Ramadas A. (2026). Behavioural and environmental determinants of overweight/obesity among Malaysian young adults: A mixed methods study using the Health Belief Model. Nutrire.

[23] Maitiniyazi G., Dilimulati M., Abulaiti R., Nijiati Z., Tuergong N., Ma T., Li C., Xia S. (2025). Clustering of lifestyle factors and the relationship with mental health among college students: A latent profile analysis. BMC Public Health.

[24] Nascimento-Ferreira M.V., Marin K.A., Abrão Ferreira R.K., Oliveira L.F., Bandeira A.C., Silva Sousa P., Miranda de Sousa J., de Almeida Cardoso A.G., Conceição da Silva L.C., Rosa A.C.A. (2022). 24 h movement behavior and metabolic syndrome study protocol: A prospective cohort study on lifestyle and risk of developing metabolic syndrome in undergraduate students from low-income regions during a pandemic. Front. Epidemiol..

[25] Onell C., Asker M., Wiberg H., Côté P., Johansson F., Sundberg T., Edlund K., Skillgate E. (2025). An unhealthy lifestyle and incident activity-limiting neck and back problems in university students: The Sustainable UNiversity Life (SUN) study. BMC Public Health.

[26] Ramón-Arbués E., Granada-López J.-M., Martínez-Abadía B., Echániz-Serrano E., Antón-Solanas I., Jerue B.A. (2021). Factors Related to Diet Quality: A Cross-Sectional Study of 1055 University Students. Nutrients.

[27] Sidebottom C., Ullevig S., Cheever K., Zhang T. (2021). Effects of COVID-19 pandemic and quarantine period on physical activity and dietary habits of college-aged students. Sports Med. Health Sci..

[28] Telleria-Aramburu N., Arroyo-Izaga M. (2022). Risk factors of overweight/obesity-related lifestyles in university students: Results from the EHU12/24 study. Br. J. Nutr..

[29] Tomás-Gallego G., Dalmau-Torres J.M., Jiménez-Boraita R., Ortuño-Sierra J., Gargallo-Ibort E. (2025). Adherence to the Mediterranean Diet in Spanish University Students: Association with Lifestyle Habits, Mental and Emotional Well-Being. Nutrients.

[30] Barbosa B.C.R., Parajára M.d.C., de Paula W., Machado E.L., Meireles A.L. (2023). Age, skin color, self-rated health, and depression associated with co-occurrence of obesogenic behaviors in university students: A cross-sectional study. Sao Paulo Med. J..

[31] Barbosa B.C.R., de Deus Mendonça R., Machado E.L., Meireles A.L. (2024). Co-occurrence of obesogenic behaviors and their implications for mental health during the COVID-19 pandemic: A study with university students. BMC Public Health.

[32] Cavalcante-Neto J.L., de Carvalho I.S., Araújo Santos K., Correia-Oliveira C.R. (2026). Associations between body image changes and modifiable behavioral variables among university students in the context of COVID-19 pandemic. Sci. Rep..

[33] Menakaya N., Menakaya I. (2022). Qualitative study exploring perceptions, attitudes and practices of adolescent university students in Lagos, Nigeria, towards a healthy lifestyle. Afr. J. Prim. Health Care Fam. Med..

[34] Zheng S., Chua X.H., Edney S.M., Goh C.M., Tai B.C., Chia J., Koek D., van Dam R.M., Müller-Riemenschneider F. (2025). Movement and dietary behaviours and mental health among university students: The Health@NUS study. BMC Public Health.

[35] West S.L., Bates H., Watson J., Brenner I.K.M. (2020). Discriminating Metabolic Health Status in a Cohort of Nursing Students: Protocol for a Cross-Sectional Study. JMIR Res. Protoc..

[36] Napolitano M.A., Lynch S.B., Mavredes M.N., Shambon B.D., Posey L. (2020). Formative work to design a digital learning self-assessment and feedback tool to prevent weight gain among college students. Digit. Health.

[37] Heller S., Reichel J.L., Mülder L.M., Schäfer M., Schwab L., Werner A.M., Letzel S., Rigotti T., Dietz P. (2024). The association between health behaviours and academic performance moderated by trait mindfulness amongst university students: An observational study. Front. Public Health.

[38] Coll Campayo I., Servera Fuster M., López-Safont N. (2026). Lifestyle, health behavior, and oral health differences among dentistry, nutrition, and fine arts university students. Front. Public Health.

[39] Pfledderer C.D., Bai Y., Brusseau T.A., Burns R.D., King Jensen J.L. (2022). Changes in college students’ health behaviors and substance use after a brief wellness intervention during COVID-19. Prev. Med. Rep..

[40] Ramírez-Díaz M.d.P., Luna-Hernández J.F., López-Cruz E., González-Jiménez A. (2023). Dietary patterns associated with physical activity and sedentary behavior in university students in Mexico. Rev. Chil. Nutr..

[41] M Y., Kagathara N., Ram R., Misra S., Kagathara J. (2023). Exploring Behavioral Risk Factors for Non-communicable Diseases Among Undergraduate Medical Students in Western Gujarat: A Cross-Sectional Study. Cureus.

[42] Thiria E., Pellegrini C., Kase B.E., DeVivo K., Steck S.E. (2024). Health behavior and anxiety changes during the COVID-19 pandemic among students, faculty, and staff at a US university. J. Am. Coll. Health J. ACH.

[43] Romero-Blanco C., Rodríguez-Almagro J., Onieva-Zafra M.D., Parra-Fernández M.L., Prado-Laguna M.D.C., Hernández-Martínez A. (2020). Physical Activity and Sedentary Lifestyle in University Students: Changes during Confinement Due to the COVID-19 Pandemic. Int. J. Environ. Res. Public Health.

[44] Monteiro L.Z., Varela A.R., de Lira B.A., Contiero L.C., Carneiro M.d.L.A., de Souza P., Nóbrega J.O.d.T., Júnior F.B. (2019). Weight status, physical activity and eating habits of young adults in Midwest Brazil. Public Health Nutr..

[45] Harmouche-Karaki M., Mahfouz M., Mahfouz Y., Fakhoury-Sayegh N., Helou K. (2020). Combined effect of physical activity and sedentary behavior on body composition in university students. Clin. Nutr..

[46] Mudarra-García N., Pérez-Mudarra M., Ortuño-Soriano I., Badía-Iborra R., Vicente-Galán M.J., Zaragoza-García I., Roque-Rojas F., García-Sánchez F.J. (2025). Impact of Exams on Diet, Physical Activity, and Body Composition in University Students. Nutrients.

[47] Woodman A., Coffey M., Cooper-Ryan A.-M., Jaoua N. (2024). The relationship between lifestyle habits and obesity among students in the Eastern province of Saudi Arabia: Using the Arab Teens Lifestyle (ATLS) questionnaire. BMC Public Health.

[48] Coppi F., Nasi M., Sabatini S., Bellini P., Generali L., Mecugni D., Farinetti A., Consolo U., Mattioli A.V. (2022). Lifestyle changes during the first and second waves of the COVID-19 pandemic in medical college students: Are there gender-related differences?. Acta Biomed..

[49] Rafraf M., Molani-Gol R., Sahebjam M. (2023). Effect of COVID-19 pandemic on eating habits and lifestyle of college students in Tabriz, Iran: A cross-sectional study. Front. Public Health.

[50] Ramón-Arbués E., Echániz-Serrano E., Martínez-Abadía B., Antón-Solanas I., Cobos-Rincón A., Santolalla-Arnedo I., Juárez-Vela R., Adam Jerue B. (2022). Predictors of the Quality of Life of University Students: A Cross-Sectional Study. Int. J. Environ. Res. Public Health.

[51] Laska M.N., VanKim N.A., Erickson D.J., Lust K., Eisenberg M.E., Rosser B.R.S. (2015). Disparities in weight and weight behaviors by sexual orientation in college students. Am. J. Public Health.

[52] Noonil N., Darakai H., Hasan M.K.B.C., Narkkul U., Kunset P. (2026). Dietary Patterns and Sociodemographic Determinants of Underweight and Excess Body Weight Among Nursing Students in Southern Thailand. Int. J. Environ. Res. Public Health.

[53] Alzamil H.A., Alhakbany M.A., Alfadda N.A., Almusallam S.M., Al-Hazzaa H.M. (2019). A profile of physical activity, sedentary behaviors, sleep, and dietary habits of Saudi college female students. J. Fam. Community Med..

[54] Alshehri M.M., Alqhtani A.M., Gharawi S.H., Sharahily R.A., Fathi W.A., Alnamy S.G., Alothman S.A., Alshehri Y.S., Alhowimel A.S., Alqahtani B.A. (2023). Prevalence of lower back pain and its associations with lifestyle behaviors among college students in Saudi Arabia. BMC Musculoskelet. Disord..

[55] Du C., Zan M.C.H., Cho M.J., Fenton J.I., Hsiao P.Y., Hsiao R., Keaver L., Lai C.C., Lee H., Ludy M.J. (2021). Health behaviors of higher education students from 7 countries: Poorer sleep quality during the COVID-19 pandemic predicts higher dietary risk. Clocks Sleep.

[56] Hutchesson M.J., Duncan M.J., Oftedal S., Ashton L.M., Oldmeadow C., Kay-Lambkin F., Whatnall M.C. (2021). Latent class analysis of multiple health risk behaviors among Australian university students and associations with psychological distress. Nutrients.

[57] Monteiro J., Cabral J., Pinto R., Pinto M.L., Santos I. (2026). Ultra-processed food consumption among university students: Application of front-of-package-based nutrient profiling scores and associations with physical activity and body composition. BMC Nutr..

[58] Troiano R.P., Berrigan D., Dodd K.W., Mâsse L.C., Tilert T., McDowell M. (2008). Physical activity in the United States measured by accelerometer. Med. Sci. Sports Exerc..

[59] Caamaño-Navarrete F., Arriagada-Hernández C., Fuentes-Vilugrón G., Guzmán-Guzmán I.P., Jara-Tomckowiack L., Jerez-Mayorga D., Del-Cuerpo I., Contreras-Díaz G., Hernández-Mosqueira C., Vargas C.A. (2025). Association between lifestyle parameters, quality of life, and satisfaction with life in Chilean university students. Healthcare.

[60] Peltzer K., Pengpid S. (2017). The Association of Dietary Behaviors and Physical Activity Levels with General and Central Obesity among ASEAN University Students. AIMS Public Health.

[61] Dun Y., Ripley-Gonzalez J.W., Zhou N., You B., Li Q., Li H., Zhang W., Thomas R.J., Olson T.P., Liu J. (2021). Weight gain in Chinese youth during a 4-month COVID-19 lockdown: A retrospective observational study. BMJ Open.

[62] Khajuria A., Yadav S.S., Somanadhapai S., Thapa R., Singh M.J., Bansal P., Vats N., Rishi N.N., Kumar L., Sharma N. (2025). Influence of dietary habits and lifestyle practices on the health and well-being of university students: A survey investigation. J. Lifestyle Med..

[63] Savage M.J., Procter E.L., Magistro D., Hennis P.J., Donaldson J., Leslie-Walker A., Jones B.A., James R.M. (2024). Characterising the activity, lifestyle behaviours and health outcomes of UK university students: An observational cohort study with a focus on gender and ethnicity. BMC Public Health.

[64] Li Y. (2024). The role of lifestyle and mental health in the weight change of higher vocational college students in Fuzhou, China during COVID-19. Prev. Med. Rep..

[65] Li Y., Liang J., Ma J., Jia Z., Li X., Su Q. (2026). Musculoskeletal pain in university students: Lifestyle determinants of prevalence and burden. Front. Public Health.

[66] Bravini E., Azzolina D., Janin D., Vercelli S., Panella M., Rinaldi C. (2022). Health-related lifestyles among Italian university students: A cross-sectional study. Epidemiol. Prev..

[67] Yoshida A. (2026). Nutritional and behavioral factors associated with the occurrence and duration of winter weight gain. Nutr. Health.

[68] Zhong Q., Huang J. (2026). Sleep, movement, and dietary behaviours: The mediating role of affect. Psychol. Health.

[69] Araya-Quintanilla F., Landaeta-Díaz L., Rosati A., Silva-Navarro C., Jeria-Díaz A., Gutiérrez-Espinoza H. (2023). Life-style behavior upon admission to higher education in Chilean university students: A longitudinal observational study. Rev. Med. Chile.

[70] Elsayed H., Alrohaily L., Alsaedi S.L., Aljohani S.M., Jan R.A., Alharthi N.N., Garah R.A. (2023). Impact of COVID-19 on the lifestyle of students of Taibah University, Madinah. Cureus.

[71] Sisay T. (2021). Physical Inactivity as a Pandemic: Daily Activities and Dietary Practices. Risk Manag. Healthc. Policy.

[72] Abdul Rahman H., Julaini N.N., Zaim S.N.N., Masri N.A., Abdul-Mumin K.H. (2023). Mental wellbeing and health-risk behaviours of university students in Brunei: A cross-sectional study during COVID-19 pandemic. Healthcare.

[73] Akter M.M., Hossain M.J. (2024). Food consumption patterns and sedentary behaviors among university students: A cross-sectional study. Health Sci. Rep..

[74] Zhang Y., Yang X., Yang Z., Chi X., Chen S. (2024). Associations of 24-hour movement guidelines adherence with fruit and vegetable intake in university students. PeerJ.

[75] Benaich S., Mehdad S., Ibn Lahmar Andaloussi Z., Boutayeb S., Alamy M., Aguenaou H., Taghzouti K. (2021). Weight status, dietary habits, physical activity, screen time and sleep duration among university students. Nutr. Health.

[76] Boukrim M., Obtel M., Lahlou L., Razine R. (2021). University students’ perceptions and factors contributing to obesity and overweigh in Southern of Morocco. Afr. Health Sci..

[77] Maté-Muñoz J.L., Hernández-Lougedo J., Ruiz-Tovar J., Olivares-Llorente R., García-Fernández P., Zapata I. (2023). Physical Activity Levels, Eating Habits, and Well-Being Measures in Students of Healthcare Degrees in the Second Year of the COVID-19 Pandemic. Healthcare.

[78] Xu X., Pu Y., Sharma M., Rao Y., Cai Y., Zhao Y. (2017). Predicting Physical Activity and Healthy Nutrition Behaviors Using Social Cognitive Theory: Cross-Sectional Survey among Undergraduate Students in Chongqing, China. Int. J. Environ. Res. Public Health.

[79] Bertrand L., Shaw K.A., Ko J., Deprez D., Chilibeck P.D., Zello G.A. (2021). The impact of the coronavirus disease 2019 (COVID-19) pandemic on university students’ dietary intake, physical activity, and sedentary behaviour. Appl. Physiol. Nutr. Metab..

[80] Schuch F.B., Waclawoscky A., Tornquist D., Oyeyemi A.L., Sadarangani K.P., Takano K., Teychenne M., Balanzá-Martínez V., O’Neil A., Romain A.J. (2026). UNIversity students’ LIFEstyle behaviours and Mental health cohort (UNILIFE-M): Study protocol of a multicentre, prospective cohort study. BMJ Open.

[81] Gutiérrez-Espinoza H., Cassola-Cajiao M., Garzón-Ulloa E., Celi-Lalama D., Bastidas-Caldes C., Araya-Quintanilla F., Cristi-Montero C., López-Gil J.F. (2024). Lifestyle behavior of physiotherapy students from Ecuador upon admission to higher education: A cross-sectional study. Front. Sports Act. Living.

