# Supplementary material for: Nutrition, Sedentary Behaviour and Physical Activity in Undergraduate Students: A Scoping Review with PRISMA-ScR Checklist
Source: Nutrients. 2026 Sep 8;18(18):2947. doi: 10.3390/nu18182947 (PMC13610651; doi:10.3390/nu18182947)
Supplement: Supplementary file 1 [file nutrients-18-02947-s001.zip › nutrients-4446807-supplementary.pdf]

Supplementary Table S1. PRISMA-ScR Checklist

Title: *Nutrition, Sedentary Behaviour and Physical Activity in Undergraduate Students: A Scoping Review with PRISMA-ScR Checklist*

Item	PRISMA-ScR Checklist Item	Reported in the Manuscript	Response
1	Identify the report as a scoping review.	Title page, page 1	The title identifies the manuscript as a scoping review.
2	Provide a structured summary including background, objectives, eligibility criteria, sources of evidence, charting methods, results, and conclusions.	Abstract, page 2	The abstract provides a structured summary including Background/Objectives, Methods, Results and Conclusions. It reports the databases searched, eligibility window, inclusion criteria, number of studies included and main findings.
3	Describe the rationale for the review in the context of what is already known.	Introduction, pages 3–4	The Introduction explains the public health relevance of dietary habits, sedentary behaviour and physical activity in undergraduate students and identifies the gap regarding studies jointly addressing these three constructs.
4	Provide an explicit statement of the questions and objectives being addressed.	Introduction, page 4	The objective is explicitly stated: to map the available evidence jointly addressing dietary habits, sedentary behaviour and physical activity among undergraduate university students, and to describe study characteristics, measurement approaches and main findings.
5	Indicate whether a review protocol exists and provide registration information if available.	Materials and Methods, page 4	No review protocol registration is reported.
6	Specify characteristics of the sources of evidence used as eligibility criteria and provide a rationale.	Section 2.2, page 5	Eligibility criteria are reported according to population, study design, publication date, language, outcomes and exclusion criteria. Eligible studies were required to include undergraduate students and to assess dietary intake or diet quality, sedentary behaviour and physical activity as distinct constructs.

7	Describe all information sources in the search and the date the most recent search was executed.	Section 2.1, pages 4–5	PubMed and Scopus were searched for English-language journal articles published between 2014 and 2026. The manuscript reports the number of records retrieved from each database.
8	Present the full electronic search strategy for at least one database, including any limits used.	Section 2.1, pages 4–5	Full search strategies are reported for both PubMed and Scopus, including search terms, Boolean operators, publication-year limits, language restrictions and document-type restrictions.
9	State the process for selecting sources of evidence.	Sections 2.3–2.4, pages 5–6; Figure 1	The manuscript describes title/abstract screening, full-text assessment, duplicate removal, handling of borderline cases and reasons for exclusion. The selection process is summarized in Figure 1.
10	Describe the methods of charting data from the included sources of evidence.	Section 2.5, page 6	Data extraction was performed independently by two reviewers using a standardized data extraction form developed a priori. Discrepancies were resolved by discussion and, when necessary, consultation with a third reviewer.
11	List and define all variables for which data were sought.	Section 2.5, page 6	Extracted variables included first author, year, country, study design, sample size, mean age, physical activity assessment, dietary assessment, sedentary behaviour assessment, intervention duration where applicable, primary outcomes and main findings.
12	If done, provide a rationale for conducting a critical appraisal and describe the methods used.	Section 4.5, page 14	No critical appraisal of individual sources of evidence was undertaken, consistent with the objectives and methodology of this scoping review.
13	Describe the methods of handling and summarizing the data that were charted.	Results, Sections 3.1–3.8; Discussion, Sections 4.1–4.5	Findings were synthesized narratively according to study characteristics, assessment tools, prevalence of unhealthy behaviours, behavioural clustering, mental health and academic outcomes, COVID-19-related findings, population heterogeneity and ongoing studies.
14	Give numbers of sources screened, assessed for eligibility and included, with reasons for exclusions at each stage.	Section 2.4, page 5; Figure 1	A total of 1,910 records were identified. After removal of 306 duplicates, 1,604 unique records were screened and 131 full-text reports were assessed for eligibility. Sixty-one full-text reports were excluded: 56 did not meet other eligibility criteria, four did not assess sedentary behaviour as a distinct construct, and one

			included an ineligible population. Seventy studies were included, comprising 62 primary studies and 8 ongoing study protocols.
15	For each source of evidence, present characteristics for which data were charted.	Table 1; Section 3.1	Table 1 summarizes the characteristics of all included studies, including author, year, country, design, sample size, age, assessment methods and main findings.
16	If critical appraisal was done, present the data.	Not applicable	Not applicable. No critical appraisal was undertaken.
17	For each included source of evidence, present the relevant data that were charted.	Table 1; Results	Relevant data from each included study are presented in Table 1 and further summarized narratively in the Results section.
18	Summarize and/or present the charting results as they relate to the review questions and objectives.	Sections 3.2–3.8; Discussion	The synthesis reports that inadequate dietary habits, insufficient physical activity and prolonged sedentary behaviour frequently coexist among undergraduate students. Sedentary behaviour emerged as the most consistently elevated construct and was often absent from otherwise relevant studies.
19	Summarize the main results, including an overview of concepts, themes and types of evidence available.	Discussion, Sections 4.1–4.4	The Discussion summarizes the main evidence, highlighting the clustering of unhealthy lifestyle behaviours, the predominance of cross-sectional self-report data, the relevance of sedentary behaviour and the scarcity of intervention studies.
20	Discuss the limitations of the scoping review process.	Section 4.5, page 14	Limitations include absence of formal critical appraisal, predominance of cross-sectional studies, heterogeneity of measurement instruments, restriction to English-language publications, strict inclusion criteria and inclusion of ongoing protocols without outcome data.
21	Provide a general interpretation of the results with respect to the review questions and objectives, as well as implications and next steps.	Section 5, pages 14–15	The Conclusion states that a broadly consistent pattern of suboptimal diet, low physical activity and elevated sedentary time was observed among undergraduate students, and that future randomized interventions should simultaneously target and measure all three behaviours.

22	Describe sources of funding for the included sources of evidence and for the scoping review.	Funding Statement, Back Matter	This scoping review received no external funding.
